# Sources of stress among long-term care workers: Findings from qualitative and survey data

**DOI:** 10.1371/journal.pone.0354970

**Published:** 2026-08-03

**Authors:** Iffath Unissa Syed, Sara Imanpour, Anita Yuskauskas, Allison Angert, George Garrow

**Affiliations:** 1 Department of Health Policy and Administration, The Pennsylvania State University, Sharon, Pennsylvania, United States of America; 2 Department of Health Administration, School of Business Administration, The Pennsylvania State University, Harrisburg, Pennsylvania, United States of America; 3 Department of Health Policy and Administration, The Pennsylvania State University, Lehigh Valley, Pennsylvania, United States of America; 4 Department of Psychology, LaFayette University, Lehigh Valley, Pennsylvania, United States of America; 5 Primary Health Network, Sharon, Pennsylvania, United States of America; Access Alliance Multicultural Health and Community Services: Access Alliance, CANADA

## Abstract

Stress is a major problem in workplaces, with connections to adverse outcomes including injuries, illness, lost-time, burnout, and turnover. Long-term care (LTC) organizations frequently report such outcomes. The purpose of this study is to investigate sources of stress among LTC workers. The research methods include: in-depth, semi-structured interviews, and a survey for quantified description. The findings indicate that time pressures, work situations, and financial situations are the major stressors identified by respondents. Respondents identified personal or family responsibilities, physical or health problems/conditions, and caring for children, as additional stressors. Many respondents established a network of friends, family, superiors, peers, and colleagues to seek social and emotional support, which served as a buffer against stress. Furthermore, respondents adopted additional coping mechanisms to combat stress, such as utilizing prayers or exercising mindfulness.

## Introduction

Stress is broadly considered to be a strain on living organisms, and is defined as non-specific responses to demands [1,2]. Stress responses are natural behavioral adaptations to a stressor to change or leave the stressors in the individual’s physical or social environment [3], and can be felt when individuals face opportunities, constraints, or demands that are perceived as uncertain yet important [4] and can manifest in both positive and negative ways. Stress is classified into two categories: distress which is harmful or disease-producing stress, and eustress, which is beneficial stress [5,6]. Negative stress can have short-term and long-term effects on the body [2, 7]. Apart from physiological impacts of stress, there are also psychological impacts of stress, such as: mood changes, negative emotions, feeling of helplessness, and behavioral impacts, the latter of which involves directly confronting the stressors [2,8].

Various words or phrases describe negative stress and its impacts, and what a lived experience of stress means to people, ranging from clinical symptoms of depression, feeling out of control or overworked, experiencing migraines or headaches, experiencing time pressure, anxiety, and sleep disturbances [9]. Stress can also impair people’s behaviors and actions in negative ways [2,10].

Job stress among healthcare workers is an increasingly common occurrence [11]. Factors that may be responsible for job stress include observing aging, morbidity, death or dying; dealing with patient’s families or relatives; and coping with the unpredictable situations that require rapid and critical decisions about interventions or treatments; and balancing work and family commitments [2].

Research suggests that there is significant job stress in particular healthcare organizations, such as Long-Term Care (LTC) environments [12]. The organizational factors that may be attributed to job stress include organizational demands for efficiency and effectiveness in health service delivery, as well as high levels of responsibility that is ascribed to workers for a large number of increasingly frail, older residents with complex needs [13]. Care work requires a variety of tasks that must be completed in a timely manner, with few resources [13,14]. In highly urbanized LTC homes, it is estimated that there are approximately 28 residents per registered healthcare worker that are responsible for residents’ complex care needs [15]. Accordingly, inherent to these front-line care-intensive occupations, there may be numerous sources of built-in stress that become occupational hazards.

Studies have shown that job-related stress is a major problem for nurse aides in LTC homes [16], and that stress results in burn-out [17,18]. There is also high turnover due to low wages [19], leadership variables from the individual (leadership practice) and organisational level (administration turnover) [20], which were found to me more important influences on turnover than personal stressors (e.g., emotional exhaustion). However, few studies have examined the sources of this stress for a wider variety of employees or residents within LTC [21,22] which would otherwise have a holistic approach to managing stress in healthcare organizations, addressing multiple stakeholders’ needs. Thus, there is a significant gap.

The purpose of this study was to fill the above knowledge gaps and examine the major sources of stress among a wide variety of workers in an urbanized LTC home in Ontario, Canada. The aim was to document these sources of stress in detail.

### The research questions asked: what are the prominent sources of stress among a broad set of employees sampled from LTC?

One of the objectives aimed to identify the factors associated with job stress, roles at work, the changing nature of relationships with other people, organizational structure and climate, domestic obligations, and work-home interface. The rationale for this was to capture factors that lead to job stress. Another objective was to analyze implications for coping, resistance, and resilience strategies. Understanding the experiences of stress and its impacts on workers’ health and wellbeing is important because it can modulate quality of care and affect the organizations’ production/service functions and efficiency.

We organize our paper with materials and methods, followed by analysis, results, discussion, strengths and limitations, and concluding remarks.

## Materials and methods

This study is derived from a larger research project that had a single-case study design, and relied on qualitative interviews, and a survey, and considered to be a mixed methods approach [23], the latter of which has higher rating for quality than case studies that rely “[…] on only single sources of information” [24]. The study was conducted in accordance with the Declaration of Helsinki, and approved by the Institutional Review Board, Office of Research Ethics (ORE) Human Participants Review Committee of York University for studies involving humans.

Recruitment occurred through in-person site visits with the assistance of a flyer posted at the entrance of the site which took place January 17, 2017 to April 28, 2017.

### Data collection

Mixed methods research design was employed in which the major sources of evidence were from face-to-face, in-depth, semi-structured interviews were conducted with 42 participants and digitally recorded. Purposeful sampling combined with snowball methods was employed. For the quantitative component, an exploratory, paper-based pilot survey was distributed to participants, as well as a separate demographic questionnaire following each interview. 92 respondents filled and returned the survey from a pool of 176 participants, i.e., a response rate of 52% was achieved. One survey was excluded as the worker was not responsible for work that was related to any aspect of the LTC home. Participants were offered incentives for their participation in the interview and for survey completion.

### Measures

For the qualitative component, multiple units of measure were organized by workers’ sex, job titles or roles, visible minority (VM) status, full time (FT) status and part-time (PT) work status, among other things. VM status was derived from participants’ responses to certain interview prompts, such as “Tell me about your background”. VM status is an important marker of difference and may be a proxy or measure of migration among particular ethnic groups/ancestral backgrounds. It is expected that coping/defense behaviors may be different depending on VM status. The survey did not explicitly ask participants to identify their “race” due to concerns that such questions could generate discomfort or anxiety among respondents. Participants may have been reluctant to respond because of concerns related to self- or group representation, fear of potential repercussions associated with disclosure, or uncertainty regarding how racialized data might be interpreted or used. Prior research involving racialized populations, including African American participants, has similarly shown that respondents may experience discomfort when disclosing racially sensitive information in research settings [25].

Instead, racialized identity was inferred through a survey item asking participants to identify their ancestral or ethnic background, with response categories including European (White), South Asian, East Asian, Aboriginal/Indigenous, African, and other backgrounds. For analytical purposes, responses were categorized into visible minority (VM)/racialized and non-visible minority (non-VM)/non-racialized groups. Participants identifying as European (White) were classified as non-VM, while all other responses were categorized as VM/racialized. Participants who selected multiple ancestral backgrounds, including at least one VM category, were also coded as VM for the purposes of statistical analysis.

For the survey, the purpose was to provide a quantified description for self-reported levels of stress using a Likert scale (1 to 5, lowest to highest interpreted from responses as not at all stressful, not very stressful, a bit stressful, quite a bit stressful, and extremely stressful, respectively), stratified by sex, VM-status, and job-categories.

### Analysis

Although qualitative and quantitative data collection was carried out concurrently, there was “separate data analysis” [26] that occurred individually. For the qualitative data analysis, fieldnotes and interview transcripts were analyzed iteratively with thematic analysis for the study using the assistance of a coding system using NVivo computer software program, to organize and sort information. During the coding and analysis process, the raw transcript text data files were imported into NVivo, and relevant text was highlighted using an initial list of codes. Themes were developed as the broader context was taken into account to find shared meanings. In the subsequent step, transcript text grouped by same code and themes were continuously compared to identify similarities and differences across participants’ narratives. Coded and thematically analyzed data were independently verified and agreed upon by collaborating researchers for intercoder reliability. Quantitative statistical data analysis of the demographic questionnaire and survey occurred by utilizing Excel, and quantitative statistical software program (IBM SPSS Statistics, 26.0, Armonk, NY). Statistical tests included Mann-Whitney U tests for comparing proportions and were deemed appropriate by independent statistical consultants given that data samples originated from a gendered sector, i.e., LTC, testing for two non-normally distributed independent groups when comparing ordinal, interval, or continuous data.

## Results

Participant characteristics from the interviews are listed in Table 1. Participant characteristics from the survey are listed in Table 2. Of the 91 survey respondents, 89 answered the question about stress. Of these respondents, 87.6% (78/89) reported at least some level of stress in their workday.

**Table 1:** Interview participants’ characteristics (n = 42).

Characteristic		Frequency	%
Sex	Female	35	83.3%
	Male	7	16.7%
	Total	42	
Employment Type	Full Time	32	76.2%
	Part Time	10	23.8%
	Total	42	
Job Title/ Role	Trainee	3	7.1%
	Allied Health	7	16.7%
	Nurse	9	21.4%
	Manager	4	9.5%
	Support Staff	6	14.3%
	Ancillary	6	14.3%
	PSW	7	16.7%
	Total	42	
Race/ VM Status	Non-VM, non-racialized, White	12	28.6%
	VM, racialized	30	71.4%
	Total	42	

**Table 2:** Descriptive statistics of the survey sample (n = 91).

Characteristic		Frequency	%
Sex	Female	76	83.5%
	Male	14	15.4%
	No response/omitted	1	1.1%
	Total	91	
Race/ VM Status	Non-VM, non-racialized	11	12.1%
	VM, racialized	78	85.7%
	No response/omitted	2	2.2%
	Total	91	
Birth/ Immigration Status	Born in Canada	19	20.9%
	Born outside Canada, i.e., Immigrant	66	72.5%
	No responses/omitted	6	6.6%
	Total	91	

**Fig 1:**
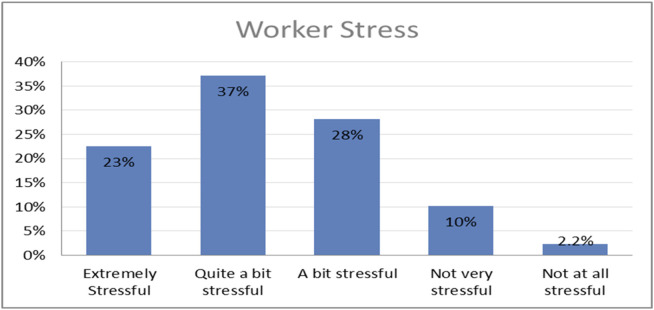
Reports of stress levels (n = 89).

22.5% (20/89) reported that their work is extremely stressful, while 37.1% (33/89) found it quite a bit stressful (Fig 1). 28.1% (25/89) reported that the work was a bit stressful. Only 10.1% (9/89) reported that it was not very stressful, while 2.2% (2/89) reported that it was not at all stressful. Overall, a majority of respondents who responded (59.6%, 53/89) reported experiencing extreme or quite a bit of stress.

Respondents were asked to elaborate on what issues contributed most to their feelings of stress. Respondents could select all the listed stressors that applied to them. Out of 91 total survey responses to this question, the top five stressors were identified as time pressures, i.e., not having enough time (74.7%, 68/91); the participant’s work situations (30.8%, 28/91); financial situations (27.5%, 25/91); personal or family responsibilities (18.7%, 17/91); and physical health problems or conditions (15.4%, 14/91) (Table 3). The fewest responses about stressors were attributed to personal or family’s safety (1.1%, 1/91); discrimination (3.3%, 3/91); personal relationships (4.4%, 4/91); health of family members (5.5%, 5/91); housing (5.5%, 5/91); or too much workload or a picky boss (6.6%, 6/91).

**Table 3:** Survey respondents: what contributes most to feelings of stress at work/ outside of work by sex. This data shows the sex distribution (frequencies and proportions) for each response about workplace stress factors/contributors of stress out of the entire cluster of men (n = 14) and women (n = 76) who participated in the survey.

	Total (n = 91)		Female (n = 76)		Male (n = 14)	
What Contributes Most to Feelings of Stress at Work/ Outside of Workϕ	Frequency	%	Frequency	%	Frequency	%
Time pressures/ not enough time*	68	74.7%	60	78.9%	7	50.0%
Own work situation*	28	30.8%	22	28.9%	5	35.7%
Financial situation	25	27.5%	19	25.0%	6	42.9%
Other personal or family responsibilities	17	18.7%	15	19.7%	2	14.3%
Own physical health problem or condition	14	15.4%	10	13.2%	4	28.6%
Caring for own children	13	14.3%	11	14.5%	2	14.3%
Employment status*	12	13.2%	6	7.9%	5	35.7%
Caring for others*	9	9.9%	5	6.6%	3	21.4%
Own emotional or mental health problem or condition	8	8.8%	6	7.9%	2	14.3%
School	7	7.7%	6	7.9%	1	7.1%
Other, e.g., too much workload, picky boss	6	6.6%	6	7.9%	0	0.0%
Housing	5	5.5%	3	3.9%	2	14.3%
Health of family members	5	5.5%	5	6.6%	0	0.0%
Personal relationships	4	4.4%	3	3.9%	1	7.1%
Discrimination	3	3.3%	2	2.6%	1	7.1%
Personal or family’s safety	1	1.1%	1	1.3%	0	0.0%

Φ Respondents could select more than one response; *1 respondent did not disclose their sex.

Stress data was stratified by sex (Tables 3 and 4). 78.9% of women (60/76) selected time pressure/not enough time as a stressor compared to 50% of men (7/14) (Table 3). Interestingly, 42.9% of men (6/14) selected financial situation as a source of stress compared to only 25% of women (19/76).

**Table 4:** Survey: particular types of stress, at work/ outside of work by sex. This data shows the sex distribution for each response about workplace stress factors/contributors of stress.

	Total (n = 91)		Female		Male	
What Contributes Most to Feelings of Stress at Work/ Outside of Work Φ	Frequency	%	Frequency	%+	Frequency	%+
Time pressures/not enough time*	68	74.7%	60	88.2%	7	10.3%
Own work situation*	28	30.8%	22	78.6%	5	17.9%
Financial situation	25	27.5%	19	76.0%	6	24.0%
Other personal or family responsibilities	17	18.7%	15	88.2%	2	11.8%
Own physical health problem or condition	14	15.4%	10	71.4%	4	28.6%
Caring for own children	13	14.3%	11	84.6%	2	15.4%
Employment status*	12	13.2%	6	50.0%	5	41.7%
Caring for others*	9	9.9%	5	55.6%	3	33.3%
Own emotional or mental health problem or condition	8	8.8%	6	75.0%	2	25.0%
School	7	7.7%	6	85.7%	1	14.3%
Other, e.g., too much workload, picky boss	6	6.6%	6	100.0%	0	0.0%
Housing	5	5.5%	3	60.0%	2	40.0%
Health of family members	5	5.5%	5	100.0%	0	0.0%
Personal relationships	4	4.4%	3	75.0%	1	25.0%
Discrimination	3	3.3%	2	66.7%	1	33.3%
Personal or family’s safety	1	1.1%	1	100.0%	0	0.0%

Φ Respondents could select more than one response; *1 respondent did not disclose their sex.

+% of frequency for that row, e.g., denominator for time pressure = 68.

Stress data was also stratified by VM/racialized status, and the findings suggest disparities in terms of mental/physical health (Table 5). For example, among those who selected particular categories of stressors, such as “own emotional or mental health problem or condition”, 100% were racialized (n=8/8), none were non-racialized (Table 6). Of those who selected “own physical health problem or condition”, 92.9% were racialized (n=13/14), and 7.1% were non-racialized (n=1/14) (Tables 7 and 8). Of those who selected “discrimination”, 100% were racialized (n=3/3), none were non-racialized (Tables 7 and 8).

**Table 5:** Survey respondents: what contributes most to feelings of stress at work/ outside of work by VM status. This data shows the distribution (frequencies and proportions) for each response about workplace stress factors/contributors of stress for the entire cluster of VMs (n = 78) and non-VMs (n = 11) who participated in the survey.

	Total (n = 91)		VM (n = 78)		Non-VM (n = 11)	
What Contributes Most to Feelings of Stress at Work/ Outside of Workϕ	Frequency	%	Frequency	%	Frequency	%
Time pressures/not enough time**	68	74.7%	56	71.8%	10	90.9%
Own work situation*	28	30.8%	24	30.8%	3	27.3%
Financial situation	25	27.5%	21	26.9%	4	36.4%
Other personal or family responsibilities	17	18.7%	16	20.5%	1	9.1%
Own physical health problem or condition	14	15.4%	13	16.7%	1	9.1%
Caring for own children	13	14.3%	11	14.1%	2	18.2%
Employment status	12	13.2%	11	14.1%	1	9.1%
Caring for others	9	9.9%	8	10.3%	1	9.1%
Own emotional or mental health problem or condition	8	8.8%	8	10.3%	0	0.0%
School	7	7.7%	5	6.4%	2	18.2%
Other, e.g., too much workload, picky boss	6	6.6%	6	7.7%	0	0.0%
Housing	5	5.5%	4	5.1%	1	9.1%
Health of family members	5	5.5%	5	6.4%	0	0.0%
Personal relationships	4	4.4%	2	2.6%	2	18.2%
Discrimination	3	3.3%	3	3.8%	0	0.0%
Personal or family’s safety	1	1.1%	1	1.3%	0	0.0%

Φ Respondents could select more than one response; *1 respondent did not disclose ancestral background.

**2 respondents did not disclose ancestral background

**Table 6:** Survey: particular types of stress at work/ outside of work by VM status. This data shows VMs’ distribution of responses for each workplace stress factors/contributors of stress.

	Total (n = 91)		VM		Non-VM	
What Contributes Most to Feelings of Stress at Work/ Outside of Work Φ	Frequency	%	Frequency	% +	Frequency	% +
Time pressures/not enough time**	68	74.7%	56	82.4%	10	14.7%
Own work situation*	28	30.8%	24	85.7%	3	10.7%
Financial situation	25	27.5%	21	84.0%	4	16.0%
Other personal or family responsibilities	17	18.7%	16	94.1%	1	5.9%
Own physical health problem or condition	14	15.4%	13	92.9%	1	7.1%
Caring for own children	13	14.3%	11	84.6%	2	15.4%
Employment status	12	13.2%	11	91.7%	1	8.3%
Caring for others	9	9.9%	8	88.9%	1	11.1%
Own emotional or mental health problem or condition	8	8.8%	8	100.0%	0	0.0%
School	7	7.7%	5	71.4%	2	28.6%
Other, e.g., too much workload, picky boss	6	6.6%	6	100.0%	0	0.0%
Housing	5	5.5%	4	80.0%	1	20.0%
Health of family members	5	5.5%	5	100.0%	0	0.0%
Personal relationships	4	4.4%	2	50.0%	2	50.0%
Discrimination	3	3.3%	3	100.0%	0	0.0%
Personal or family’s safety	1	1.1%	1	100.0%	0	0.0%

Φ Respondents could select more than one response; *1 respondent did not disclose ancestral background

**2 respondents did not disclose ancestral background

+% of frequency, e.g., time pressure = 68

**Table 7:** Survey: What Contributes Most to Feelings of Stress at Work/ Outside of Work by Job Category and Subgroup (continues on next page). This data shows the occupational distribution (frequencies and proportions) for each response about workplace stress factors/contributors of stress out of the entire cluster of PSWs (n = 34), Nurses (n = 19), Allied Health workers (n = 10), Ancillary workers (n = 10), Support Staff (n = 9), Managers (n = 5) and Trainees (n = 4) who participated in the survey.

	Total (n = 91)		PSW (n = 34)		Nurse (n = 19)		Allied health (n = 10)	
What Contributes Most to Feelings of Stress at Work/ Outside of WorkΦ	Frequency	%	Frequency	%	Frequency	%	Frequency	%
Time pressures/ not enough time	68	74.7%	25	73.5%	17	89.5%	5	50%
Own work situation	28	30.8%	9	26.5%	7	36.8%	4	40%
Financial situation	25	27.5%	9	26.5%	3	15.8%	4	40%
Other personal or family responsibilities	17	18.7%	6	17.6%	4	21.1%	2	20%
Own physical health problem or condition	14	15.4%	3	8.8%	2	10.5%	1	10%
Caring for own children	13	14.3%	3	8.8%	2	10.5%	5	50%
Employment status	12	13.2%	1	2.9%	5	26.3%	2	20%
Caring for others	9	9.9%	2	5.9%	4	21.1%	1	10%
Own emotional or mental health problem or condition	8	8.8%	1	2.9%	1	5.3%	2	20%
School	7	7.7%	0	0.0%	1	5.3%	2	20%
Other, e.g., too much workload, picky boss	6	6.6%	3	8.8%	2	10.5%	0	0%
Housing	5	5.5%	3	8.8%	0	0.0%	1	10%
Health of family members	5	5.5%	2	5.9%	0	0.0%	2	20%
Personal relationships	4	4.4%	1	2.9%	1	5.3%	1	10%
Discrimination	3	3.3%	1	2.9%	1	5.3%	0	0%
Personal or family’s safety	1	1.1%	0	0.0%	1	5.3%	0	0%

Φ Respondents could select more than one response.

**Table 8:** Survey: what contributes most to feelings of stress at work/ outside of work by job category and subgroup (continued from previous page).

	Ancillary (n = 10)		Support Staff (n = 9)		Manager (n = 5)		Trainee (n = 4)	
What Contributes Most to Feelings of Stress at Work/ Outside of WorkΦ	Frequency	%	Frequency	%	Frequency	%	Frequency	%
Time pressures/ not enough time	8	80.0%	5	55.6%	4	80%	4	100%
Own work situation	4	40.0%	3	33.3%	1	20%	0	0%
Financial situation	4	40.0%	2	22.2%	1	20%	2	50%
Other personal or family responsibilities	1	10.0%	2	22.2%	1	20%	1	25%
Own physical health problem or condition	4	40.0%	2	22.2%	1	20%	0	0%
Caring for own children	1	10.0%	1	11.1%	1	20%	0	0%
Employment status	4	40.0%	0	0.0%	0	0%	0	0%
Caring for others	0	0.0%	1	11.1%	1	20%	0	0%
Own emotional or mental health problem or condition	2	20.0%	1	11.1%	1	20%	0	0%
School	0	0.0%	0	0.0%	1	20%	3	75%
Other, e.g., too much workload, picky boss	1	10.0%	0	0.0%	0	0%	0	0%
Housing	0	0.0%	1	11.1%	0	0%	0	0%
Health of family members	0	0.0%	1	11.1%	0	0%	0	0%
Personal relationships	1	10.0%	0	0.0%	0	0%	0	0%
Discrimination	1	10.0%	0	0.0%	0	0%	0	0%
Personal or family’s safety	0	0.0%	0	0.0%	0	0%	0	0%

An interesting finding was that, those who selected particular stressors, tended to be women (especially racialized women), such as stress due to: “health of family members” was selected by five racialized respondents, all five being women; and “personal or family’s safety” was selected by a single racialized respondent, who was a woman (Tables 3,4,6–8). Other stressors included: “Other…workload, picky boss” which was selected by six racialized respondents, all six being women. The absences of non-VM and men as respondents for these variables reflect gendered and racialized ways in which women carry out not only paid care work, but also unpaid, domestic care work, and family responsibilities.

Stress data was also stratified by job category and may reflect socioeconomic status disparities between particular groups of workers (i.e. PSWs vs. other categories of workers in terms of financial situation and housing) (Tables 7–10). For example, “housing” was not a major source of stress (5.5%, 5/91). However, of those who selected “housing”, 60% (n=3/5) were PSWs; 20% (n=1/5) were allied health workers, and 20% (n=1/5) were support staff. That is, for workers with high socioeconomic status, such as managers and nurses, housing is not a source of stress.

**Table 9:** Survey: particular types of stress at work/ outside of work by job category (continues on next page). This data shows the occupational distribution for each response about workplace stress factors/contributors of stress.

Total (n = 91)			PSW		Nurse		Allied health	
What Contributes Most to Feelings of Stress at Work/ Outside of WorkΦ	Frequency	%	Frequency	% +	Frequency	% +	Frequency	% +
Time pressures/ not enough time	68	74.7%	25	36.8%	17	25.0%	5	7.4%
Own work situation	28	30.8%	9	32.1%	7	25.0%	4	14.3%
Financial situation	25	27.5%	9	36.0%	3	12.0%	4	16.0%
Other personal or family responsibilities	17	18.7%	6	35.3%	4	23.5%	2	11.8%
Own physical health problem or condition	14	15.4%	3	21.4%	2	14.3%	1	7.1%
Caring for own children	13	14.3%	3	23.1%	2	15.4%	5	38.5%
Employment status	12	13.2%	1	8.3%	5	41.7%	2	16.7%
Caring for others	9	9.9%	2	22.2%	4	44.4%	1	11.1%
Own emotional or mental health problem or condition	8	8.8%	1	12.5%	1	12.5%	2	25.0%
School	7	7.7%	0	0.0%	1	14.3%	2	28.6%
Other, e.g., too much workload, picky boss	6	6.6%	3	50.0%	2	33.3%	0	0.0%
Housing	5	5.5%	3	60.0%	0	0.0%	1	20.0%
Health of family members	5	5.5%	2	40.0%	0	0.0%	2	40.0%
Personal relationships	4	4.4%	1	25.0%	1	25.0%	1	25.0%
Discrimination	3	3.3%	1	33.3%	1	33.3%	0	0.0%
Personal or family’s safety	1	1.1%	0	0.0%	1	100.0%	0	0.0%

Φ Respondents could select more than one response; + % of frequency count, e.g., time pressure n = 68.

**Table 10:** Survey: particular types of stress at work/ outside of work by job category (continued from previous page).

	Ancillary		Support Staff		Manager		Trainee	
What Contributes Most to Feelings of Stress at Work/ Outside of WorkΦ	Frequency	% +	Frequency	% +	Frequency	% +	Frequency	% +
Time pressures/ not enough time	8	11.8%	5	7.4%	4	5.9%	4	5.9%
Own work situation	4	14.3%	3	10.7%	1	3.6%	0	0.0%
Financial situation	4	16.0%	2	8.0%	1	4.0%	2	8.0%
Other personal or family responsibilities	1	5.9%	2	11.8%	1	5.9%	1	5.9%
Own physical health problem or condition	4	28.6%	2	14.3%	1	7.1%	0	0.0%
Caring for own children	1	7.7%	1	7.7%	1	7.7%	0	0.0%
Employment status	4	33.3%	0	0.0%	0	0.0%	0	0.0%
Caring for others	0	0.0%	1	11.1%	1	11.1%	0	0.0%
Own emotional or mental health problem or condition	2	25.0%	1	12.5%	1	12.5%	0	0.0%
School	0	0.0%	0	0.0%	1	14.3%	3	42.9%
Other, e.g., too much workload, picky boss	1	16.7%	0	0.0%	0	0.0%	0	0.0%
Housing	0	0.0%	1	20.0%	0	0.0%	0	0.0%
Health of family members	0	0.0%	1	20.0%	0	0.0%	0	0.0%
Personal relationships	1	25.0%	0	0.0%	0	0.0%	0	0.0%
Discrimination	1	33.3%	0	0.0%	0	0.0%	0	0.0%
Personal or family’s safety	0	0.0%	0	0.0%	0	0.0%	0	0.0%

### Qualitative data/ interview findings

The interview findings reveal that stress among long-term care (LTC) workers is shaped by a combination of emotional labor, excessive workloads, difficult resident care demands, and unsupportive organizational environments. Across occupational groups, participants described stress not only as an individual experience but also as a structural issue linked to management practices, staffing pressures, and workplace culture. At the same time, workers emphasized the importance of faith, mindfulness, social support, and personal routines as coping strategies. These findings support the research argument that occupational stress in LTC settings is multidimensional and deeply connected to organizational conditions.

One trainee commented about the emotional contradiction of LTC work by explaining that although caregiving is meaningful, it is also psychologically demanding:

“[This] is a rewarding field, but it can also be very stressful. I chose to work in this profession because I want to make a difference in other people’s lives as well as my own. My love for medicine has also pushed me to become a [job title]. My religion, fitness, and well-balanced diet are all the things that keep me going so that I can maintain a healthy body and mind”. (Respondent #027, Trainee, Female, VM, workload status unknown)

This quote is significant because it supports and contextualizes the quantitative results and also captures the dual nature of care work—simultaneously fulfilling and emotionally exhausting. It also demonstrates how workers rely on holistic coping strategies, including religion and self-care, to sustain themselves in stressful environments. This supports the argument that stress management in LTC extends beyond workplace interventions and includes personal resilience practices.

Most interview participants repeatedly reported experiencing moderate stress levels associated with their work at this LTC facility, citing organizational failures, particularly poor management and unrealistic expectations. One nurse stated:

“[…] I think the managers or just management with this place puts a lot of stress on the workers mentally […]the expectation that they have for us is ridiculous, and good communication method is not the best.” (Participant 7, Nurse, Female, VM, P/T).

This perspective is important because it shifts attention away from stress as merely an individual burden and toward institutional causes of burnout. The participant identifies managerial practices and communication breakdowns as major contributors to workplace distress, reinforcing the argument that organizational culture significantly shapes worker well-being.

Another participant connected stress to workload intensity and supervisory pressure, explaining, “[T] he boss will give you a shit if you don’t finish the project. Yeah. Anyway. What to do?” (Participant 12, Ancillary Worker, Male, VM, F/T).

Although brief, this statement powerfully illustrates how workplace hierarchies and pressure from supervisors intensify stress, particularly for ancillary workers whose labor is often overlooked in LTC research. This evidence supports the broader argument that stress affects multiple occupational categories within LTC facilities, not only frontline nursing staff.

The strongest evidence of emotional exhaustion emerged in a nurse’s account of burnout:

“I am so burned out here. This place will mentally destroy you and I just want to get out of here. But I can’t because of the money”

The above finding is especially significant because it demonstrates the severe psychological consequences of chronic workplace stress, including emotional entrapment and loss of professional fulfillment. The participant’s inability to leave due to financial dependence signifies how economic precarity compounds occupational stress. This finding strongly supports the argument that LTC burnout is systemic rather than individual, rooted in unsustainable working conditions.

The same nurse indicated further explained that workers felt unsupported when dealing with challenging resident behaviors:

“[…] You go to the management; you tell them ‘I have a behavior.’ They tell you to deal with it. They don’t help you medicate; they don’t help you talk to the doctor.” (Participant 17, Nurse, Female, VM, F/T).

The above statement is important because it illustrates workers’ perceptions of abandonment by management and the absence of institutional support systems. It reinforces the argument that inadequate organizational responses exacerbate emotional strain and contribute to burnout and depression among LTC staff.

Stress was also linked to experiences of disrespect and bullying. A support staff described how management failed to defend her after an incident with a visitor:

“[…] I didn’t realise that this place does not implement rules. The rules are there, and management don’t care about rules. You can do whatever you want.” (Participant 28, Support Staff, Female, VM, P/T).

This participant’s experience is significant because it highlights how workers experience stress not only through workload but also through feelings of injustice, lack of protection, and organizational neglect. The account demonstrates how workplace stress is intensified when employees feel unsupported by leadership.

Finally, another participant emphasized the growing demands associated with aging residents:

“[T]hey’re [the residents are] getting old plus when you’re dealing with some residents [who] are very demanding. They want to take all your time for them and it’s not possible.” (Participant 34, PSW, Female, VM, F/T).

The above finding is important because it situates stress within the realities of increasing care complexity in LTC settings. It supports the argument that demographic and clinical changes among residents place additional emotional and physical demands on workers, often without corresponding increases in staffing or institutional support.

Given that participants demonstrated a variety of sources of stress in interviews, the need to examine the various coping mechanisms to reduce stress is elicited.

Research suggests mindful breathing, meditation, prayer, walking, listening to music, and swimming; and practicing self-care as ways to manage stress [27]. Other methods include negative coping (maladaptive behaviors) such as smoking, consumption of alcohol (ibid). In addition to these techniques, LTC workers draw upon social support and vent to family, friends, and the community (ibid), and engage in solitary recreation, and take short vacations, outings, and trips (ibid).

### Coping strategies

Participants were questioned about the use of various coping mechanisms to reduce stress in the workplace. Many participants indicated that good coping mechanisms help combat stress and enable them to control their emotions and mental health from depression and burnout. Participants claimed that they do not have specific stress management approaches to reduce stress in the workplace. The prime steps in coping with job stress were to take constructive actions in reducing job stress such as tackling the root cause of job stress, talking-out problems with relatives or friends, learning to relax during stressful events, and involvement in non-work activities.

This study shows that participants frequently referenced faith, mindfulness, prayers and social support to cope with stress. A nurse explained:

“[S] speaking to God about your concerns or your struggles or going to church and speaking to our pastor about currently what’s going on to seek guidance, […] I try and seek social support like around me, or reach out further wherever I need to.” (Participant 10, Nurse, Female, Non-VM, F/T).

This finding is significant because it demonstrates that coping is both spiritual and relational. Workers relied on family, faith communities, and peers to process stress and maintain emotional balance. It also supports the argument that informal support networks are essential protective factors against burnout in LTC environments.

Similarly, another worker described a simple but meaningful nightly coping ritual which included prayer:

A: “After I go from here, I won’t talk to anybody. Take shower, pray, then I go to the bed.” (Participant 36, PSW, Female, VM, F/T).

Although concise, this finding reflects how workers create routines of emotional decompression after stressful shifts. Its significance lies in illustrating how coping strategies are embedded in everyday practices that help workers psychologically separate themselves from workplace stress.

Overall, the interview findings demonstrate that LTC workers experience stress through intersecting pressures related to workload, resident care demands, managerial practices, workplace conflict, and emotional exhaustion. At the same time, participants’ reliance on faith, social support, mindfulness, and personal rituals highlights the importance of coping mechanisms in sustaining worker resilience. Together, these findings support the broader research argument that occupational stress in LTC facilities is both structurally produced and individually negotiated through diverse coping practices.

### Mixed-methods analysis and joint interpretation

The qualitative interview findings deepened and contextualized our statistical patterns by illustrating how workers experience stress in their everyday lives. Interview participants repeatedly described excessive workloads, understaffing, unrealistic managerial expectations, difficult resident behaviors, and lack of organizational support as major stressors, which further expands upon survey responses for “time pressures/not enough time” “own work situation” “employment status” and “caring for others”.

The integrated findings demonstrate important occupational and socioeconomic differences in stress experiences. Quantitative results indicate that occupational stress is closely linked to broader social determinants of health, including gender, race, immigration, and socioeconomic position. Qualitative findings reinforced this interpretation by showing how workers felt trapped in stressful environments because of economic necessity and supported the survey results that workers in lower-paid occupational groups experienced greater socioeconomic stress.

Gendered and racialized dimensions of stress also emerged across both data strands. Survey findings showed that racialized women disproportionately reported family-related and workload-related stressors. The interview findings complemented these results by revealing how many racialized female workers simultaneously managed emotionally demanding care work, domestic responsibilities, and expectations of self-sacrifice. This convergence suggests that stress among LTC workers cannot be understood solely through workplace factors, but must also be interpreted within broader systems of gendered and racialized labor inequality.

The mixed-methods integration further demonstrated convergence regarding organizational culture and leadership. Quantitative findings identified work situations and workload pressures as primary stressors, while qualitative findings clarified that workers frequently associated these pressures with inadequate communication, lack of managerial support, bullying, and weak enforcement of workplace policies. The qualitative accounts therefore explain the mechanisms underlying the survey trends by illustrating how organizational dysfunction translates into emotional exhaustion, frustration, and burnout.

Interview narratives provide further explanatory depth to the survey results by demonstrating that stress is not merely an individual psychological experience, but rather a structural and organizational phenomenon rooted in labor-intensive working conditions, hierarchical workplace relationships, and inadequate institutional support.

At the same time, both data sources highlighted the importance of coping and resilience strategies. Survey results suggested that workers relied on social and emotional support systems to manage stress. Qualitative findings elaborated on these patterns by showing that workers frequently used prayer, mindfulness, exercise, social support, and personal rituals as coping mechanisms. One participant described seeking guidance through prayer and conversations with family and peers, while another explained that her nightly routine involved showering, praying, and isolating herself after work to decompress emotionally. These findings indicate that coping occurs at multiple levels—individual, relational, spiritual, and community-based—and serves as an important protective factor against burnout.

Importantly, the integrated analysis reveals a tension between individual coping and structural stress production. Although workers demonstrated resilience through mindfulness, spirituality, and social support, the qualitative findings suggest that these strategies often function as survival mechanisms rather than solutions to the root causes of stress. The quantitative prevalence of stress combined with interview accounts of burnout and emotional exhaustion indicate that coping strategies alone are insufficient without broader organizational and policy-level interventions.

Overall, the joint interpretation demonstrates strong convergence between the quantitative and qualitative findings. The quantitative results establish the widespread prevalence and demographic patterning of stress, while the qualitative findings explain the lived experiences, meanings, and mechanisms underlying those patterns. Together, the integrated findings support the broader conclusion that stress among LTC workers is structurally produced through demanding labor conditions, organizational failures, and intersecting social inequalities, while coping strategies reflect workers’ ongoing efforts to maintain emotional wellbeing and resilience within highly challenging care environments.

## Discussion

Research suggests that various workplace factors contribute to stress, including understaffing, office politics, job insecurity, and lack of control over work pace [28–30]. Other research [31] indicated that work overload, uncooperative patients, excessive criticism, and lack of support from supervisors and hierarchical relationships with colleagues resulted in stress among hospital nurses, leading to poor job performance. In addition, uncertainty in the job (such as role ambiguity and role conflict), organizational stress, intensity (speed of work), and job insecurity (danger of unemployment, lack of leisure time) were among the job stressors most experienced in the workplace [32]. The physical work environment, including crowded workspaces, strong odors, interruptions/disruptions, and physical strain can also be potential sources of stress in the workplace [8,9,33–35].

The evidence from this study demonstrates several important points. Firstly, it demonstrates the workers’ dynamic responsibilities, and deep levels of involvement/ commitment to their duties, even though their work (both unpaid and paid) is often labor-intensive, highly stressful, and negatively impacts their health and wellbeing. This study supports the idea that balancing work/life is important, and home-work life conflict can be a source of stress if work interferes with an individual’s ability to fulfill home and family obligations [36,37]. One explanation for this is that caregiving can be a source of multiple role conflicts as well as guilt [38]. Furthermore, personal life events like marriage, illness, and financial worries significantly contribute to stress [39], thus, understanding the interplay between work, home, and personal life is crucial for addressing stress effectively.

Secondly, the analysis indicates that workers are also drawing upon support from a variety of sources, including their co-workers. Thus, this study demonstrates that supportive relationships are essential for work. The lack of supportive relationships or poor relationships with peers, colleagues and superiors are potential sources of stress [39–41]. Relationships in the workplace can include relationships with superiors, colleagues, subordinates, clients/consumers who use goods/services, and suppliers. The top causes of job stress include job demands, especially involving interactions with ‘demanding’ people [39].

Finally, while the workers are remunerated for their care work in the site of study, it should be noted that many LTC workers must rely upon their own resources for support in order to deal with the hazards stemming from their stressful, labor-intensive positions [42]. The analysis also revealed cultural/religious importance among respondents, demonstrating the ways in which workers exercise resistance/resilience to life circumstances by utilizing strategies such as self-care, spirituality, recreation with family or friends, and solitary recreation.

It is expected that these unique findings will help to address the ways in which healthcare organizations manage employee stress and improve workers’ health, safety, and wellbeing. The goal is to cope or minimize the extent of stress, thereby avoiding burnout and permanent health-limiting impacts, which also impair employee performance [2,43].

### Strengths and limitations

This study was limited methodologically because it was cross-sectional rather than longitudinal, and it was also limited in generalizability as it is limited to one LTC facility and utilized non-probability sampling such as purposeful/snowball sampling, which could result in selection bias, including non-representative samples, and could limit the context by making findings applicable to only certain subgroups or the immediate-convenience-based context rather than the broader population. There were also small subsamples to the investigation of job stress and coping mechanisms among health care workers, which made subgroup comparisons unstable. We caution that these results should not be overinterpreted given the small sizes. Another limitation is that for the survey data, some measures were taken from validated instruments such as the Canada Community Health Survey and further modified/developed by the PI, thus acknowledging having limited construct validity. In addition, physicians were not included in this study, which could have added further insight from another professional perspective.

Underutilization of skills, knowledge, and experiences is also a source of stress, [44] which was not assessed, but, may be important, as workers may try to get their foot in the job market by taking on underpaid work, such as that of caregivers, which can lead to exploitation [42], and could be an area of further exploration in the future.

Stress is harmful if it is continuous, as it is directly associated with increased blood pressure, heart rate, coronary heart disease, sweating, hot and cold spells, breathing difficulty, muscular tension and increased gastrointestinal disorders/ stomach ulcers, influence on sleep, mood, and social satisfaction [45], which were worse on rest days following night shifts. or the onset of other types of illness, engaging negative behaviors/activities [46–48] such as increased anger, anxiety, depression, lowered self-esteem, poorer intellectual functioning, inability to concentrate and make decisions, nervousness, irritability, higher alcohol and other drug abuse, impulsive behavior resentment of supervision and job dissatisfaction [49]. Stress is implicated in the incidence and development of mental illness, certain types of cancer, smoking, dietary problems, excessive alcohol consumption and substance abuse, life dissatisfaction, accident and unsafe behavior at work, migraine, hay fever, asthma and skin rashes, marital and family problems [37,50]. Moreover, it was identified that an individual who has poor problem-solving and coping skills, inability to understand and cope with own emotions, and lack of social and self-assertion skills is more vulnerable to stress and subsequent alcohol and drug misuse [51].

Stress is also problematic from a business, economics, and organizational behavior perspective, as it results in decreased performance, absenteeism, higher accident rates, higher turnover rates as well as resentment of supervision and job dissatisfaction [49]. Researchers in one study found that nurses often desire work absence due to tiredness, health issues, and work-life interference [52]. Other work [53] on nurses’ job stress found role conflict and role overload negatively affect job performance and motivation and correlated with absenteeism and turnover. Overall, stress is a ubiquitous aspect of life, distinguished by eustress and distress. Eustress can motivate individuals to excel, while distress undermines performance and disrupts adaptive functioning [54].

This work highlights the need for effective coping strategies [55–57]. Coping behaviors are non-automatic, adaptive processes [58], encompassing actions to control or reduce stress effects [59,60], and ultimately, coping entails managing challenges, expending effort to solve problems, and reducing stress [38,61,62]. Social support, recognized as a valuable coping mechanism, can reduce stress [63,64]. It involves seeking reassurance, understanding root causes of stress, and maintaining a sense of control [65] as well as fostering friendly, trustworthy relationships within one’s social network [66]. Supportive partners and friends can aid in problem-solving during stressful events [2,67]. In order to achieve effective and positive coping behaviors, anticipating and preparing appropriate plans of action should be undertaken (ibid). Social support has been recognized and proved to be an excellent coping mechanism to reduce stress [63,64].

Social support can be defined as a trustworthy and cooperative exchange between members of the social network to which an individual belongs [66–68], as they provide information, guidance, practical aid, and emotional support with positive effects on stress reduction [69]. Management might also want to increase worker participation in decision-making, WHICH fosters communication among workers and improves interpersonal relations, leading to increased social support and reduced job-related strain [70]. Job demands and coping skills influences health outcomes, with high coping and low demands correlating with fewer health problems [71].

Coping strategies favoring escape or only focusing on symptom-management or a misplaced search for cures as opposed to targeting the source of the stress may cause psychosomatic complaints [57]. Contrarily, problem-solving coping is associated with higher performance under stress [72,73]. Gender differences in coping responses exist, with women generally exhibiting higher levels of control coping and social support [74], which should also be kept in mind when implementing positive coping strategies in organizations. Worker involvement in stress interventions is crucial for success [75], with targeted resources and organizational interventions essential for stress reduction [76].

It would be useful to have more research that extends the sampling of other staff. In addition, research suggests that shift work is a source of physical and mental stress [9,77,78], and increases incrementally as the number and length of night shifts increase [79]. The evidence from this study does not adequately shed light in this area, and more research would be required to see if shift work induces significant stress among LTC workers. Directing attention to invisible structural factors such as poverty, distress, duress, and under-nutrition, might also be insightful, as they would highlight the underlying issues of income inequality, social exclusion, colonialism, and racism that also need to be addressed [80–83].

## Conclusions

The findings suggest that while there are a variety of coping, resistance, and resilience practices, particular behavioral mechanisms are encouraged. At the macro policy level, health behavior change is the primary focus, and there is an emphasis on the benefits of population health management through exercise and diet interventions. At the meso-policy community and health care institution level, there was a focus on organizational health and human resource factors, which were manifested in seeking social and community resources. At the micro-policy individual level, personal coping health behaviors were emphasized. Future studies may offer additional insights into this sector for a variety of additional workers.

## Abbreviations

LTC: long term care workers
